# Conserved Non-Coding Regulatory Signatures in *Arabidopsis* Co-Expressed Gene Modules

**DOI:** 10.1371/journal.pone.0045041

**Published:** 2012-09-14

**Authors:** Jacob B. Spangler, Stephen P. Ficklin, Feng Luo, Michael Freeling, F. Alex Feltus

**Affiliations:** 1 Department of Genetics and Biochemistry, Clemson University, Clemson, South Carolina, United States of America; 2 Plant and Environmental Sciences, Clemson University, Clemson, South Carolina, United States of America; 3 School of Computing, Clemson University, Clemson, South Carolina, United States of America; 4 Department of Plant and Microbial Biology, University of California, Berkeley, California, United States of America; University of California, Los Angeles, United States of America

## Abstract

Complex traits and other polygenic processes require coordinated gene expression. Co-expression networks model mRNA co-expression: the product of gene regulatory networks. To identify regulatory mechanisms underlying coordinated gene expression in a tissue-enriched context, ten *Arabidopsis thaliana* co-expression networks were constructed after manually sorting 4,566 RNA profiling datasets into aerial, flower, leaf, root, rosette, seedling, seed, shoot, whole plant, and global (all samples combined) groups. Collectively, the ten networks contained 30% of the measurable genes of *Arabidopsis* and were circumscribed into 5,491 modules. Modules were scrutinized for *cis* regulatory mechanisms putatively encoded in conserved non-coding sequences (CNSs) previously identified as remnants of a whole genome duplication event. We determined the non-random association of 1,361 unique CNSs to 1,904 co-expression network gene modules. Furthermore, the CNS elements were placed in the context of known gene regulatory networks (GRNs) by connecting 250 CNS motifs with known GRN *cis* elements. Our results provide support for a regulatory role of some CNS elements and suggest the functional consequences of CNS activation of co-expression in specific gene sets dispersed throughout the genome.

## Introduction

Complex gene interactions control biological processes and a detailed knowledge of their underlying regulatory mechanisms is critical to understand, repair, and manipulate biological organisms. A powerful technique for modeling massive gene product interaction systems is the construction of a gene interaction network [1]. A gene interaction network graph is an intuitive construct that consists of nodes (gene products), non-random dependencies between genes (edges), and annotation of nodes and edges (attributes). While built from simple components, the biological network is capable of modeling tens of thousands of gene relationships in a well-defined mathematical environment suitable for higher order exploration such as coordinated gene function and regulation inference derived from network topology [2], [3].

A specific class of gene interaction network, the co-expression network, describes gene interaction as the non-random correlation of steady-state RNA output between genes. Coordinately expressed gene sets tend to implement common biological function and should impart similar gene regulation mechanisms (e.g. [4]). Through a meta-analytical approach, numerous groups have mined large, mixed-condition gene expression datasets to construct networks and to partition the network into co-expressed gene clusters (modules) underlying complex biological activities [5], [6], [7], [8], [9]. A co-expressed gene module elucidated under defined experimental conditions (e.g. tissue source, treatment conditions, genetic background, etc.) can be viewed as the end product of context-specific gene regulatory network pathways [10]. Therefore, the co-expression network is a powerful tool to explore the functional output of dependent genes as well as identify common (and possibly complex) mechanisms of coordinated gene regulation.

Steady-state RNA transcript output from genes is known to be regulated through a variety of mechanisms including transcriptional and post-transcriptional mechanisms [11]. For example, *cis*-acting DNA elements such as transcription factor (TF) binding sites [12] and miRNA target motifs [13] interact with *trans*-acting factors activated under discrete temporal and spatial conditions and coordinate enhancement or repression of target gene output [12]. In plants for example, the *cis*-acting drought response element (DRE; A/GCCGAC) recruits *trans*-acting DRE-binding proteins (DREB) that affect gene expression in response to abiotic stress [14], [15]. A specific collection of *cis* and *trans* regulatory factors compile a gene regulatory network (GRN), which Mejia-Guerra *et al* defined as “composed of transcription factors (TFs) and microRNAs (miRNAs), *trans* factors that regulate transcription or RNA translation/degradation, via *cis*-elements in the promoters of their target genes or in their resulting mRNAs respectively” [16]. GRN elucidation is an active area of research in all organisms, and a collection of validated and putative *Arabidopsis* GRNs can be found in the *Arabidopsis* Gene Regulatory Information Server (AGRIS) database AtRegNet; [10]. Through the non-random assignment of *cis* regulatory motifs to GRN target genes in co-expression network modules, it is possible to associate one or more GRNs as the potential mediators of co-expression network topology.

A potentially profound influence on the formation of gene co-expression relationships is gene duplication in which coding sequences and flanking regulatory DNA is multiplied, providing a new source of genetic information for selection [17]. Multiple modes of gene duplication occur, frequent and rare, in all multicellular organisms including tandem, whole-genome, segmental, and transposition events [18]. In the *Arabidopsis thaliana* (hereafter *Arabidopsis*) lineage there have been three whole genome duplication events, with the most recent being a dramatic tetraploidy event occurring ∼23.2 Mya (alpha duplication event) [19], [20], [21]. Remnants of the alpha event can be detected in the form of duplicate open-reading frames (alpha duplicates) and proximal conserved non-coding DNA sequences (CNSs; [22]) that have resisted deletion (fractionation) over millions of years of evolution. Clearly, many of these DNA patterns that have been copied and conserved should contain functional information including gene regulatory potential.

We hypothesized that CNS elements detected in remnants of the alpha event are involved in the regulation of steady state mRNA levels in *Arabidopsis*. In support, *Arabidopsis* CNS elements have been shown to influence both co-expression and expression intensity of alpha duplicate pairs in *Arabidopsis* and that CNS regulatory mechanisms may be a combination of transcriptional and post-transcriptional control [23]. In this study, we sought evidence for a regulatory role of CNS elements in the formation of co-expression relationships in alpha duplicate genes as well as genes found elsewhere in the genome. Our primary goal was to determine the non-random association of CNS elements with tissue sorted co-expression network gene modules. A CNS-enriched module can be hypothesized to be under partial *cis* control by the CNS, and once placed into the context of known GRNs provides a working model for the complex regulation that created a co-expressed gene set. In this study, we were able to determine hundreds of functionally annotated gene modules from tissue-enriched co-expression networks and provide evidence that many are controlled by CNS-encoded regulatory mechanisms.

## Results

### Construction of *Arabidopsis* Co-expression Networks

In order to maximize detection of co-expression relationships relevant to specific tissues and organs, we used 4,566 *Arabidopsis* Affymetrix® ATH1 microarray samples, obtained from the NCBI Gene Expression Omnibus database [24], that were previously subdivided by manual curation into nine tissue-enriched datasets: aerial, flower, leaf, root, rosette, seedling, seed, shoot, and whole plant (whole) [23]. Nine co-expression networks were then constructed from these presorted groups which we termed: Aerial, Flower, Leaf, Root, Rosette, Seedling, Seed, Shoot, and Whole networks. A tenth Global network was constructed using all 4,566 microarray expression samples. Expression dataset input our network construction pipeline ranged in size from 108 samples (Seed) to 877 (Leaf) samples. Significant pairwise correlations for each network were determined using the random matrix theory (RMT) hard threshold method [25] with significant correlation thresholds ranging from 0.836 (Seedling) to 0.942 (Seed) (Table 1). The node count for each tissue-enriched network varied from 800 nodes (Shoot) to 1,780 (Aerial), accounting for 3.9% to 8.6% of the measureable gene space on the microarray platform. The frequency of genes unique to a tissue-enriched network ranged from 9.6% (Seedling) to 49.4% (Flower), while the unique edge count (co-expression relationships) ranged from 38.3% (Seedling) to 83.0% (Seed) (Table S1). When combined, the number of unique genes present in the nine tissue-enriched networks was 5,947, or 28.8% of the measurable genes. The Global network contained 95,004 edges and 2,606 nodes, representing 12.6% of measurable genes of the array platform (Table 1). The total number of unique genes in the ten networks was 6,246, representing 30.2% of the measurable *Arabidopsis* gene space.

**Table 1:** Arabidopsis Co-expression Network Properties.

Network	Arrays	Nodes	Edges	<k>	PCC	MCL	LCM
Aerial	231	1,780	5,217	5.9	0.862	342	278
Flower	146	972	8,043	16.5	0.941	113	187
Leaf	877	920	4,553	9.9	0.902	148	181
Root	640	1,690	9,537	11.3	0.837	297	323
Rosette	268	1,627	5,867	7.2	0.864	285	289
Seedling	675	1,722	13,562	15.8	0.836	261	435
Seed	108	1,081	3,574	6.6	0.942	201	177
Shoot	305	800	4,699	11.7	0.926	119	172
Whole	771	1,735	17,111	19.7	0.851	211	426
Global	4,566	2,606	95,004	72.9	0.487	236	810

<k>  =  Average connectivity;

PCC  =  Pearson correlation coefficient significance threshold;

MCL  =  Markov clustering modules;

LCM  =  Link community modules.

Each of the ten networks was then subdivided into modules of inter-connected genes using the Markov Cluster (MCL) and link communities methods (Table S2) [26], [27]. We refer to the genes in link communities as Link Community Modules (LCM). The MCL algorithm circumscribes mutually exclusive modules whereas the LCM method allows for node overlap between modules. The number of MCL modules per network ranged from 113 (Flower) to 342 (Aerial) while the number of LCM modules ranged from 172 (Shoot) to 810 (Global) (Table 1). The MCL algorithm assigned all nodes to modules and captured 68.0% (Aerial) to 95.1% (Global) of the network edges. The LCM algorithm captured 59.8% (Aerial) to 93.8% (Global) of the network edges and 43.8% (Aerial) to 66.0% (Flower) of network nodes. In total, 5,491 modules were detected across all ten networks.

### Significant Association of CNS Elements with Co-expressed Gene Modules

CNS elements were previously detected as conserved sequence patterns proximal to alpha duplicate gene pairs [22] and may play a role in the co-regulation of alpha duplicate gene pairs [23]. Functional CNSs contain information (regulatory and otherwise) that was copied during the whole genome duplication event and resisted deletion, presumably through the selective advantages associated with maintenance of the duplicate gene pair as opposed to fractionation. Any function encoded in a CNS element could be active elsewhere in the genome which would simply be missed in the CNS screen that was focused on proximal alpha duplicate gene space. Therefore, we sought to evaluate CNS regulatory patterns outside of alpha duplicate genes by identifying CNS elements in non-alpha duplicates across the *Arabidopsis* genome. CNS elements that were found near fractionated (non-alpha) genes were termed CNS’ elements. In total 10,439 out of 11,452 CNS elements were identified in close proximity to 18,853 genes throughout the genome (Table S3). Thus, we assigned 56.1% of *Arabidopsis* genes (TAIR10 build) with a CNS’ element compared to 11.2% unfractionated alpha genes near CNS elements.

Co-expression edges represent statistically dependent relationships. We hypothesized that co-expressed genes on an edge or within an LCM or MCL module share common regulatory features that are the correlation source. Specifically, we hypothesized that co-expressed genes share GRN components, including *cis* regulatory DNA elements (CREs), that may be encoded in CNS or CNS’ elements. To address this, we tested if network modules were A) enriched in genes (nodes) that contain CNS or CNS’ elements; or B) demonstrated the non-random occurrence of co-expressed gene pairs (edges) that share the same CNS or CNS’ element, which were termed shared regulatory edges (SREs).

First we evaluated all MCL and LCM modules for significant enrichment of genes proximal to CNS or CNS’ elements that contain putative CREs, an indicator that the module might be regulated by the CRE. Starting with MCL modules, the number of unique enriched CNS elements varied from 25 (Flower) to 107 (Aerial) while the number of unique enriched CNS’ elements was slightly higher ranging from 32 (Flower/Shoot) to 123 (Aerial; Table 2). Enrichment within the Global network MCL modules was similar with 54 CNS and 92 CNS’ enriched elements. Combining enrichment results for all of the 2,213 MCL modules resulted in 411 CNS and 549 CNS’ enriched elements (Bonferroni p≤0.001; Table S4). Within LCM modules, the number of unique enriched CNS elements varied from 29 (Shoot) to 92 (Whole), while the number of unique enriched CNS’ elements ranged from 22 (Shoot) to 58 (Root; Table 2). Enrichment within LCM modules in the Global network was high relative to the nine tissue-enriched networks with 105 CNS and 91 CNS’ detected elements. Combining enrichment results for all 3,278 LCM modules resulted in 329 CNS and 360 CNS’ enriched elements (Bonferroni p≤0.001; Table S4). All enriched CNS or CNS’ elements were then examined for uniqueness to a network, a potential indicator of tissue-specific control. On average, 36% of CNS elements and 58% of CNS’ elements enriched in modules were exclusive to a given network (Table 2). In total, module enrichment revealed 1,288 CNS or CNS’ elements enriched in 1,830 modules across all networks.

**Table 2:** Unique Regulatory Elements in Co-expression Network Modules.

	CNS		CNS’		CNS in SRE		CNS’ in SRE	
Network	*MCL*	*LCM*	*MCL*	*LCM*	*MCL*	*LCM*	*MCL*	*LCM*
Aerial	107 (59)	52 (27)	123 (80)	52 (35)	10 (10)	34 (34)	38 (15)	39 (0)
Flower	25 (8)	44 (24)	32 (21)	43 (30)	12 (12)	0 (0)	12 (0)	48 (20)
Leaf	45 (10)	47 (10)	45 (28)	26 (14)	0 (0)	2 (2)	0 (0)	28 (0)
Root	84 (51)	49 (11)	96 (64)	58 (30)	20 (12)	22 (20)	0 (0)	40 (12)
Rosette	78 (37)	69 (24)	87 (45)	53 (37)	20 (10)	23 (23)	9 (1)	56 (30)
Seedling	65 (20)	69 (10)	69 (26)	41 (20)	6 (6)	20 (18)	52 (10)	48 (20)
Seed	68 (34)	44 (27)	88 (53)	51 (33)	6 (0)	24 (24)	8 (0)	36 (20)
Shoot	35 (7)	29 (8)	32 (22)	22 (14)	4 (2)	22 (16)	17 (0)	22 (4)
Whole	69 (21)	92 (20)	74 (42)	45 (22)	10 (10)	24 (20)	38 (14)	36 (10)
Global	54 (17)	105 (38)	92 (29)	91 (46)	105 (87)	41 (35)	114 (59)	75 (31)

MCL  =  Markov clustering modules; LCM  =  Link community modules; SRE  =  Shared regulatory edge; CNS  =  Conserved noncoding sequence.

Numbers in parentheses represent regulatory element frequency specific to corresponding network.

Next we used permutation testing to identify modules with a non-random occurrence of SREs. Starting with MCL modules with a significantly higher number of SREs relative to background, the number of CNS elements varied from 0 (Flower) to 34 (Aerial) while the number of CNS’ elements tended to be higher ranging from 22 (Shoot) to 56 (Rosette; Table 2). Within the Global network MCL modules, a significant number of SREs ranged higher for CNS (41) and CNS’ (75). Combining results for all of the 2,213 MCL modules resulted in 202 CNS and 216 CNS’ enriched elements (Bonferroni p≤0.001; Table S5) from modules with a significant number of SREs. Within LCM modules the number of CNS elements in modules with a significant number of SREs varied from 0 (Leaf) to 20 (Root/Rosette), while CNS’ elements tended to be higher ranging from 0 (Leaf/Root) to 52 (Seedling; Table 2). The Global network was high compared to the nine tissue-enriched networks with 105 CNS and 114 CNS’ elements. Combining results for all of the 3,278 LCM modules resulted in 169 CNS and 154 CNS’ elements (Bonferroni p≤0.001; Tables S5). In total, SRE permutation testing identified 469 unique CNS or CNS’ elements in 165 modules with a significant number of SREs across all networks. Enriched elements in modules with significant proportions of SRE were also screened for network exclusivity. On average, 81% of CNS elements and 26% of CNS’ elements found were exclusive to each network (Table 2). The existence of exclusively enriched CNS and CNS’ element across tissue-enriched networks suggests the possibility of tissue-specific function, which was not considered further in this study. After combining node enrichment and SRE significance results, we were able to assign 1,361 unique CNS or CNS’ elements to 1,904 modules.

### Mapping CNS Elements to Gene Regulatory Networks (GRNs)

While individual genes can be regulated by a single *cis*-regulatory module (CRM) [28], we expected that co-expressed modules were likely the result of complex regulation through multiple CREs and CRMs which may be acting in one or more GRNs [28], [29]. To place the CNS and CNS’ elements into a known regulatory network context, we first mapped module genes to known *Arabidopsis* GRN target genes from the AtRegNet GRN database [10]. On average, for all ten networks, 24.8% of the modules contained genes of known GRN targets, with an average of 2.4 targets per module (Table S6). Next, we tested whether these putative CNS/CNS’-embedded CREs overlapped with AtRegNet GRN-CREs. To do this, we mapped each of the 471 unique GRN-CREs collected from AtRegNet to the CNS or CNS’ elements and found that 250 of the unique CNS/CNS’-embedded CREs contained known GRN-CREs (Table S6). The remaining 1,111 CNS/CNS’ CREs were not represented in the AtRegNet database, and our results provide evidence for their role as novel GRN components. Interestingly, only 26 of the 1,904 modules mapped to CNS or CNS’ elements contained nodes annotated as transcription factors (TFs) indicating that TFs are rarely co-expressed with putative regulatory targets in a module (Table S6).

## Discussion

These results support the hypothesis that CNS elements are involved in the regulation of steady state mRNA levels in *Arabidopsis*. We provide evidence in the form of non-random association of CNS and CNS’ elements with co-expression network modules indicating a regulatory role of CNS-encoded CREs beyond alpha duplicate genes and into the broader genome. Specifically, we provide evidence of *cis* regulatory function for 1,361 unique CNSs across 1,904 co-expressed gene modules. A CNS−/CNS’-enriched module can be hypothesized to be under partial *cis* control by the element. Moreover, when these elements were placed into the context of known gene regulatory networks (GRNs), a model was created of the complex regulation underlying a co-expressed gene module. Furthermore, our method filtered insignificant CNS and CNS’ elements that are either non-functional (artifacts?), weakly involved in coordinated expression of module genes, or are not involved in mechanisms that control steady state RNA levels.

A current limitation of global co-expression networks is that many gene interactions are often missed because of mixing transcriptome states which confounds the detection of diluted but relevant relationships. This may confound the detection of genes controlled by overlapping GRNs and CREs such as the CNS elements examined in this study. Significance thresholding of pairwise expression correlations ensures that networks contain highly-significant, non-random interactions [30]. However, if a treatment condition or tissue source is underrepresented in an expression profile collection, then a real interaction relevant to that cellular environment could be masked and remain undiscovered. The end result is that global co-expression networks often capture a small portion of the measurable RNA interactome of an organism. For example existing rice, maize and *Arabidopsis* co-expression networks captured between 10 to 20% of the measurable genes respectively [5], [7], [31], [32]. This implies that assignment of coordinated gene output to relevant biological function is incomplete and the data mining potential of public databases is not fully realized. Through manual pre-sorting of expression data into tissue-enriched groups, our network collection increased capture of *Arabidopsis* genes in co-expression relationships to 30.2% enhancing the power to detect diluted tissue-specific gene interactions.

Previous co-expression networks have been constructed from grouped samples designed for a specific test [7] or focused on select tissues of interest [8]. Our approach gathers all available expression data for a holistic view of co-expression, and attempts to reduce noise created by mixing disparate datasets via partitioning samples into ontology defined expression sets. The composite of all nine tissue-enriched networks captured 5,947 unique nodes (28.7% of the measurable gene space), 51,750 unique edges, and 1,977 (MCL)/2,468 (LCM) modules. This was a marked improvement over the Global network, which captured 12.6% of the measurable gene space. The sample size of each tissue network is in line with the prescribed “optimum” of 100 microarrays [33], and each network contained overlapping and distinct regulatory information (Table 2). Therefore, these networks and their functionally annotated modules are a significant improvement in the description of the *Arabidopsis* interactome.

The network partitioning algorithm played an important role in our ability to detect putative CRE-encoded CNS/CNS’ elements in modules. Each algorithm (MCL vs. LCM) found distinct differences in node-based enrichment for CNS (411 vs. 329) and CNS’ (549 vs. 360) elements (Table S4). We expected the total number enriched elements in LCM modules to be lower as LCM modules only captured an average of 50.0% of the nodes in tissue-enriched networks. This was supported in that LCM modules captured 0.23 unique elements per module on average compared to 0.47 unique elements per MCL module. Notably only 25% (334) of the node enriched CNS or CNS’ elements were found in both MCL and LCM modules. It should be noted that SRE-based association of CNS signatures to modules was also different for each module set (MCL vs. LCM): CNS (202 vs. 169) and CNS’ (216 vs. 154) elements. This suggests that both node-based and edge-based CRE to module association approaches could be used in conjunction with alternate module discovery techniques to maximize the detection of potential module-CRE relationships.

**Figure 1:**
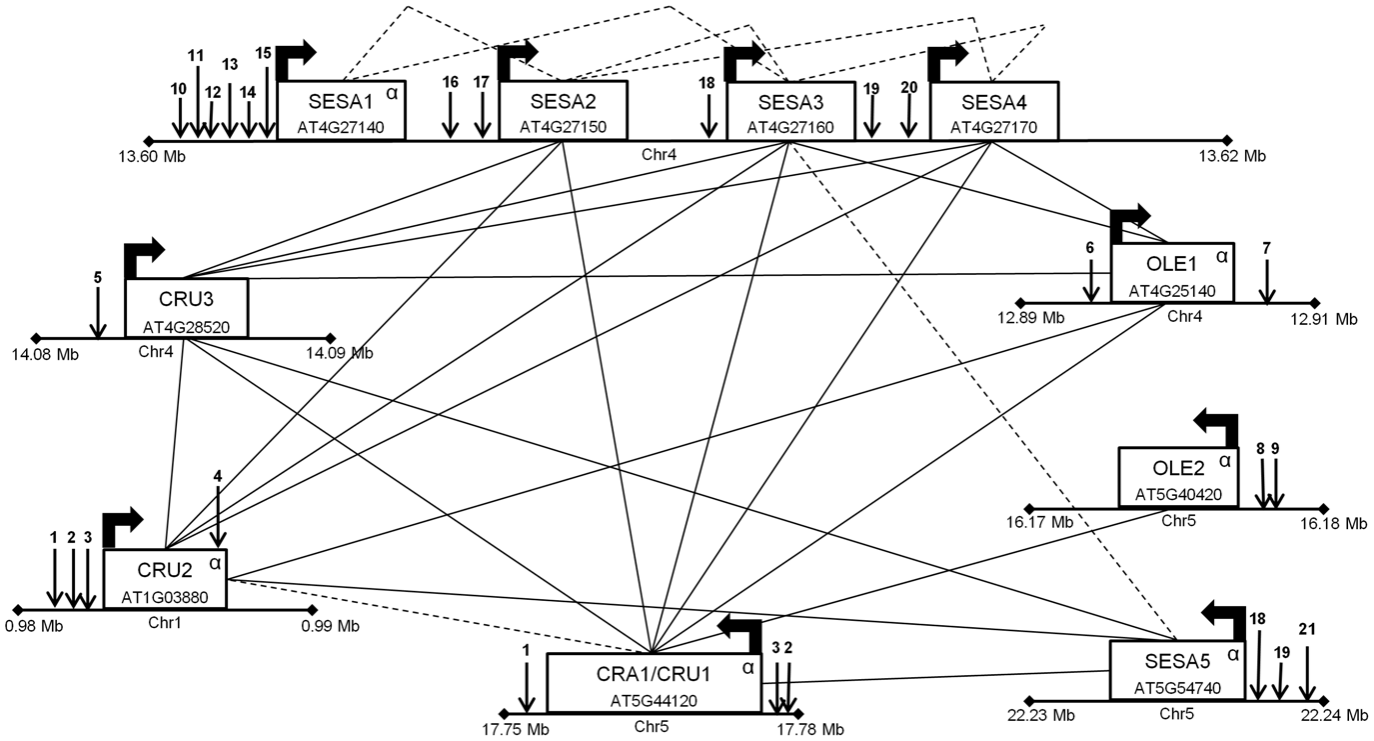
Module Aerial-MCL25. Aerial-MCL25 represents a module with a significant proportion of shared regulatory edges (SRE) and the highest number of enriched CNS’ elements. SREs are found between genes in close proximity as well as genes on different chromosomes. All genes are shown with their approximate coordinates within the *Arabidopsis* genome (e.g. SESA1, SESA2, SESA3 and SESA4 are tandem duplicates on chromosome 4 at 13.60 Mb). Most genes shown are involved in seed growth and development. Alpha duplication genes have been designated with the symbol α. Bent black arrows represent the direction of gene transcription. Black downward arrows represent CNS’ elements and unique elements are identified by different numbers. Solid black lines represent co-expression network edges and black dotted lines are shared regulatory edges (SRE). (CRU  =  Cruciferin; OLE  =  Oleosin; SESA  =  seed storage albumin).

For each module annotated with putative CREs in our study (Table S6), evidence is provided for the regulation of that gene set. For example, Aerial-MCL25, which contained the largest number of enriched CNS’ elements (9), was comprised of 10 genes that group into three families: three Cruciferins [34], two Oleosins [35] and five seed storage albumins genes (SESA; [36], [37]) (Figure 1 and Table 3). Four of the five SESA genes exist in tandem on chromosome four (SESA1, SESA2, SESA3 and SESA4) and seven of the ten genes share CNS’ elements (CRU1, CRU2, SESA1, SESA2, SESA3, SESA4 and SESA5; Figure 1). Seven of the module’s twenty-seven edges exist between genes that share CNS’ elements (CNS’ SRE), although only two of these edges exist between genes that are not part of the tandem SESA block (SESA3-SESA5 and CRU1-CRU2; Figure 1). Many of these genes are also co-expressed in other MCL modules across the nine tissue-enriched networks (Seedling, Seed, Shoot and Whole; Table S2), suggesting that their co-expression relationships are robust across temporal and spatial conditions. In addition, some of the enriched CNS’ elements for the Aerial-MCL25 module contain basic leucine zipper (bZIP) and MYB transcription factor binding sites, which have been associated with seed storage proteins (Table 3; [8]). The combination of CNS’ elements encoded for known CRE motifs and those without known function provides a framework for the regulatory analysis of this example module, a representative model for each module identified in our network collection.

**Table 3:** Regulatory Element Enrichment within Network Module Aerial-MCL25.

Element	p-value^A^	FDR^B^	Known CRE Motifs
CNS_007564	3.51E-09	7.64E-11	GTNNAC; G-box; bHLH/MYB; bZIP
CNS_007554	6.32E-09	1.44E-10	GTNNAC; G-box; ARR; bHLH/MYB; bZIP
CNS_007560	6.32E-09	1.40E-10	ABRE; E-box; G-box; DPBF; MYC
CNS_007562	2.85E-06	7.71E-08	–
CNS_007563	2.85E-06	7.92E-08	–
CNS_007558	3.06E-04	1.02E-05	–
CNS_006699	5.10E-04	1.89E-05	phyA
CNS_006700	5.10E-04	1.96E-05	phyA
CNS_007556	5.10E-04	1.82E-05	GTNNAC; G-box; ARR; bHLH/MYB; bZIP

A Bonferroni corrected p-value;

B FDR  =  False Discovery Rate;

CRE  =  cis-regulatory DNA element.

Was the regulatory potential captured by CNSs more likely to be maintained in unfractionated parts of the genome? We tested this by counting CNS’ occurrences in close proximity to alpha duplicate genes versus the remainder of the genome. The proportion of alpha duplicate genes with CNS’ elements was found to be significantly higher compared to non-alpha duplicate genes (p<0.00001). Eighty percent (5,076) of alpha duplicate genes were assigned at least one CNS’ element compared to 51% (13,777) of non-alpha genes. In comparison, the propensity of CNS elements to resist fractionation may be duplication-mechanism specific since only 4% of transposed genes in *Arabidopsis* have annotated CNSs [38]. This suggests that CNSs encode regulatory potential that favors retention after whole genome duplication events.

In conclusion, our co-expression network collection provides an extended model of the *Arabidopsis* RNA interactome for the discovery of gene regulatory mechanisms. Specifically, we applied this model to test the hypothesis that CNS elements encode regulatory information affecting the co-expression of both alpha duplicates and genes found in fractionated genome regions. In support of this hypothesis, we found that over 34% (1,904) of co-expressed gene modules were significantly associated with CNS or CNS’ elements. In addition, we identified 1,111 putative CRE-encoded CNS signature, extending known GRN models. These data demonstrate the utility of gene co-expression networks for deepening our view into the *Arabidopsis* regulome.

## Methods

### *Arabidopsis* Co-expression Network Construction

All microarray expression datasets in this study were comprised of the nine ontology sets after normalization and quality control, as described in [39] (Table S7). All networks were generated by constructing a similarity matrix of pairwise Pearson correlations for every probe set across all samples. A random matrix theory (RMT) based algorithm [30] was used to select a hard threshold of significant correlation. All probe sets in the RMT-thresholded networks were then mapped to genes using ATH1 mappings available via TAIR [40] (affy_ATH1_array_elements-2010-12-20.txt; ftp://ftp.arabidopsis.org/home/tair/Microarrays/Affymetrix/). Of the original 22,810 probe sets on the ATH1 platform, all Affymetrix control probe sets (prefixed with AFFX), probe sets that did not map to a gene model in TAIR10 (non-genic), probe sets that mapped to multiple loci (ambiguous), or probe sets that were shared by a single gene (redundant) were removed (Table S8). The final count of probe sets used was 20,677. After probe set filtering, modules were generated using the Markov Cluster algorithm (MCL; [26]). MCL modules were generated using the clustermaker v1.1 plugin with Cytoscape v2.82 using default parameters (inflation value = 2.0) (http://www.cgl.ucsf.edu/cytoscape/cluster/clusterMaker.html; http://www.cytoscape.org/). LCM modules were identified with the linkcomm [41] package in R (binary version 1.0-4; http://cran.r-project.org/web/packages/linkcomm/index.html). Module assignments for all genes within networks can be found in Table S2.

### Genome Screening for CNS elements

All of the 11,452 TAIR8-derived CNS sequences [22], [23] were aligned using BLASTN against the TAIR10 chromosomes masked for coding and repeat sequences. TAIR10 chromosomes and coding sequences were downloaded from TAIR (ftp://ftp.arabidopsis.org/home/tair/Sequences/Whole_chromosomes/chr.fas; ftp://ftp.arabidopsis.org/home/tair/Sequences/blast_datasets/TAIR10_blastsets/TAIR10_cds_20101214). *Arabidopsis* repeat sequences were downloaded from the MSU database (ftp://ftp.plantbiology.msu.edu/pub/data/TIGR_Plant_Repeats/TIGR_Arabidopsis_Repeats.v2_0_0.fsa) All BLAST hits were limited to an e-value of a 15/15 exact base pair match (e-value ∼ 0.2). BLAST results were then filtered for alignments of at least 90% of the original CNS sequence length before being considered CNS’ sequences. CNS’ sequences were assigned to all genes within 2000 bp (upstream or downstream) using a Perl script. This resulted in 10,439 unique CNS’ sequences assigned to 18,853 genes (Table S3).

### CNS/CNS’ Element Enrichment within Modules

All modules in the ten networks were tested for enrichment of CNS or CNS’ regulatory element terms using a DAVID-like [42] functional profiling strategy using in house Perl scripts [5], [43]. All terms were tested for enrichment across all networks and network modules via a Fisher’s exact test using a Perl script. Any terms with a Bonferroni p-value ≤0.001 were considered significantly enriched (Table S4).

### Shared Regulatory Edge Enrichment

All networks were separated into groups of edges completely contained within modules (intramodule) and edges that existed between modules (intermodule). Using a Perl script intramodule edges with shared CREs (CNS, CNS’) between both nodes were identified. These edges were referred to as shared regulatory edges (SRE). Modules with more than one edge and a count of one or more SRE were tested for a significant proportion of SREs by randomly selecting the same edge count from the background of all network edges (intermodule and intramodule edges) 10,000 times. The p-values were obtained by dividing the number of instances in which permuted SRE counts were higher than observed SRE counts across all permutations (Table S5).

### AtRegNet GRN-module Associations

Module genes were mapped to the ‘TargetLocus’ in AtRegNet (reg_net_20100915.tbl) downloaded from http://arabidopsis.med.ohio-state.edu. A list of Transcription Factor Binding Sites (TFBS) active at the transcriptional level was obtained from the AtRegNet AtcisDB (http://arabidopsis.med.ohio-state.edu/AtcisDB/), which comprised 471 unique TFBS elements dispersed across the *Arabidopsis* genome [44]. *Cis* elements from AtRegNet were aligned to CNS or CNS’ elements via blastn [45] and filtered for 100% sequence identity over 100% of the shortest aligned sequence, a word score of 5 and a minimum e-value of 100. The collection of 1,926 transcription factor genes was obtained from the supplemental data in [22]. Primary gene descriptions and symbols for TAIR10 can be found in Table S9.

### Enrichment of Functional Terms within Modules

All modules in the ten networks were tested for enrichment of CNS or CNS’ regulatory element terms using a DAVID-like [42] functional profiling strategy using in house Perl scripts [5], [43]. All terms were tested for enrichment across all networks and network modules via a Fisher’s exact test using a Perl script. Any terms with a Bonferroni p-value ≤0.001 were considered significantly enriched (Table S4). All GO (ATH_GO_GOSLIM.txt; ftp://ftp.arabidopsis.org/home/tair/Ontologies/Gene_Ontology; 10-25-2011) and Interpro (TAIR10_all.domains; ftp://ftp.arabidopsis.org/home/tair/home/tair/Proteins/Domains/; 11-18-2010) annotations were downloaded from TAIR. All TAIR10 peptide sequences (TAIR10_pep_20101214.txt) were downloaded from ftp://ftp.arabidopsis.org/home/tair/Proteins/TAIR10_protein_lists and submitted to the KEGG Automatic Annotation server on 10-26-2011 [46]. Enrichment of functional terms including gene ontology (GO), protein domains (Interpro), and biochemical pathways (KEGG) within all modules can be found in Table S4.

## Supporting Information

Table S1 **Unique Edge and Node Frequency Across Networks.**
(XLSX)

Table S2 **Gene Assignments to MCL/LCM Modules.**
(XLSX)

Table S3 **CNS’ Assignments to Genes.**
(XLSX)

Table S4 **Enriched Function Annotation and Regulatory Element Signatures in MCL/LCM Modules (Bonferroni p-value < = 0.001).**
(XLSX)

Table S5 **Co-enrichment of Regulatory Elements Within Modules and Module Edges.**
(XLSX)

Table S6 **Extending AGRIS GRNs to Network Modules.**
(XLSX)

Table S7 **GEO Experiment Assignment to Each Tissue-enriched Network.**
(XLSX)

Table S8 **ATH1 Probe Set/TAIR Locus ID Mapping Counts.**
(XLSX)

Table S9 **Primary Gene Descriptions and Symbols for TAIR10.**
(XLSX)
